# Predicting postnatal depressive symptoms in a prospective cohort study in Rwanda: the impact of poor maternal social support

**DOI:** 10.3389/fgwh.2023.1113483

**Published:** 2023-07-21

**Authors:** Providence M. Umuziga, Darius Gishoma, Michaela Hynie, Laetitia Nyirazinyoye, Etienne Nsereko

**Affiliations:** ^1^ Department of Mental Health Nursing, College of Medicine and Health Sciences, School of Nursing and Midwifery, University of Rwanda, Kigali, Rwanda; ^2^ Department of Psychology, York University, Toronto, ON, Canada; ^3^ School of Public Health, College of Medicine and Health Sciences, University of Rwanda, Kigali, Rwanda; ^4^ School of Health Sciences, College of Medicine and Health Sciences, University of Rwanda, Kigali, Rwanda

**Keywords:** postnatal depression, maternal social support scale, edinburgh postnatal depression scale, mental health, Rwanda

## Abstract

**Background:**

Postnatal depression is a significant public health issue that demands attention, and recent evidence indicates that rates are relatively high in low-income countries such as Rwanda. However, lack of social support is recognized as a potential risk factor for postnatal depressive symptoms. This study sought to explore the influence of poor maternal social support on postnatal depressive symptoms in a sample of women in Rwanda.

**Method:**

A prospective cohort research design was conducted with women recruited from four different health centers in Rwanda's Southern Province. A sample of 396 pregnant women accessing antenatal care services was recruited at the baseline from their late second term or later, then followed up after giving birth. The dropout rate was 21.46%; thus, the data of 311 women were analyzed. The outcome variable was the presence of depressive symptoms (Edinburgh Postnatal Depression Scale (EPDS) (≥12 cut-off score), while predictor variables included maternal social support measured using a modified Maternal Social Support Scale (MSSS), perceived health status, socio-demographic information (marital status, wealth class, age, education, occupation), negative life events, gestational and obstetric information (parity, pregnancy intention, age at birth, children given birth, and mode of delivery). Univariate and multivariate analyses were performed.

**Results:**

From a sample of 311 participants, over a quarter (20.9%) had elevated postnatal depressive symptoms (EPDS ≥ 12 scores). Elevated scores were predicted by poor perceived health status; respondents reporting neither poor nor good (AOR* *=* *0.28, CI = 0.11; 0.72, *p *= 0.007) or good health (AOR* *=* *0.14, CI = 0.05; 0.37, *p *= 0.001) were less likely to be affected. Poor maternal social support was also linked with postnatal depressive symptoms; poor partner support (AOR* *=* *4.22; CI = 1.44; 12.34; *p *= 0.009) was associated with high risk, while good friend support (AOR = 0.47, CI = 0.23; 0.98, *p *= 0.04) was a significant protector. Additionally, violence or negative life events were also independent predictors of postnatal depressive symptoms (AOR: 2.94, CI: 1.37–6.29, *p *= 0.005).

**Conclusion:**

Postnatal depressive symptoms were found to affect one in five Rwandan women. However, good maternal social support can be a strong protector. Early interventions targeting mothers in the postnatal period and strengthened social support networks for women at risk should be developed.

## Background

Depression is projected to become the second most prevalent general health condition globally (1). It poses a significant public health concern, particularly among childbearing women (2). The postnatal period, encompassing the time from childbirth to one year after delivery involves substantial psychological adaptations for women who have given birth. The postpartum period, specifically the 6–8 weeks following childbirth, involves processes that restore the mother to her pre-pregnancy state, such as the regression of the uterus and postpartum healing (3). However, this normal state can potentially expose postpartum women to an increased risk of mood swings. Postnatal depression (PND) is influenced by various sociodemographic, psychological, and obstetric factors (4). Sociodemographic and psychological risk factors for PND include previous depressive episodes, anxiety and despair during pregnancy, low self-esteem, poor marital relations, poverty, and isolation (5). Lack of social support has also been associated with PND (6–8). Additionally, caregiving stress (9, 10) and body image dissatisfaction after birth (11) can contribute to the development of postnatal depressive symptoms. Obstetric factors such as risk of perinatal complications, hospitalization during pregnancy, or cesarean delivery (12) also increase the likelihood of PND. In low- and middle-income countries (LMICs), PND is influenced by cultural norms, infant gender, marital issues, and economic hardship (13, 14). Finally, infant characteristics such as prematurity, low birth weight, or newborn dysfunction may also elevate the risk of PND (15).

Globally, approximately 13%–19% of postnatal women experience depression (8). The risk of depression is 5.7% in the second postnatal month and 5.6% in the sixth, while the lifetime risk is 10%–25% (16). The risk of depression is typically assessed through the use of standardized tools that measure depressive symptomology. Symptoms of PND include anxiety, sadness, sleep problems, difficulties concentrating and with appetite, lack of interest in the baby and surroundings, as well as suicidal ideation (5, 17, 18). Consequently, PND can impact the mother-child bond (19).

Quantifying the prevalence of PND symptoms in low-income countries is challenging due to inadequate screening, diagnosis, and underreporting by healthcare providers (3). However, research utilizing the Edinburgh Postnatal Depression Scale (EPDS) suggests a prevalence of 19.8% of individuals with elevated depressive symptoms in LMICs (20). Elevated depressive symptoms affects females in Africa throughout the postnatal period, with studies using the EPDS indicating a pooled prevalence of PND at 16.84 (17). In Azale and colleagues' population-based research in Ethiopia, one out of every ten postnatal mothers have symptoms of PND (21). A healthcare facility-based study in Uganda discovered a very high incidence of PND symptoms among postnatal women (27.1%) (22). However, despite its prevalence, PND is understudied and undertreated in Africa (17, 23). This is also the case in Rwanda, where a high prevalence of PND symptoms has also been reported. In a recent study, depressive symptoms were found to affect twice as many women in the postnatal period as in the prenatal period (24). An unpublished study in a community-based sample in Rwanda, indicated that 22% of postnatal women were affected by depressive symptoms (14). The aim of this study was to gain a greater understanding of PND in Rwanda by exploring the influence of maternal social support on postnatal depressive symptoms in a community sample.

## Material and methods

### Research design, setting and population

This study employed a prospective cohort research design, conducted in four different health centres located in Rwanda's Southern Province. Pregnant women in their late second term or beyond, receiving antenatal care services at selected health facilities and voluntarily agreeing to participate in the study, were recruited at baseline from June to July 2019. These individuals were then followed up after giving birth. The study obtained ethical approval from the Institutional Review Board (IRB) at the College of Medicine and Health Sciences (CMHS), University of Rwanda (UR).

### Sample size determination and sampling procedure

The sample size was determined using G*Power software (25). The calculated sample size was 74 participants per group, assuming an effect size of.05, error probability 0.05, a power (1-error probability) of 0.95, and 4 groups. An additional 26 participants were added to each group to account for potential attrition. All participants aged 15 years or older, attending antenatal care services at four randomly selected health centres, and in their sixth month of pregnancy or beyond, were invited to participate in the study with the help of a midwife/nurse in charge of ANC services. Community health workers in charge of maternal and new born care from selected health centres assisted in identifying eligible individuals who did not have scheduled appointments during the data collection period. A total of 396 pregnant women were initially recruited, but due to attrition, the final evaluation included 311 postnatal women (78.54%).

### Data collection instruments

A structured questionnaire was used to gather data on socio-demographic information, risk and protective variables, gestational and obstetric information, and perceived health status. The research instrument included standardized tools to assess postnatal depressive symptoms and maternal social support.

To detect postnatal depressive symptoms, the Edinburgh Postnatal Depression Scale (EPDS) was used. The EPDS has been widely used in LMICs (26). In sub-Saharan Africa, the EPDS has been validated as a reliable tool for assessing both the severity and the likelihood of depression during the perinatal period in various countries and languages (27). An unpublished study of EPDS diagnostic validity with 200 women utilizing postnatal services in Rwanda found the EPDS to have an optimal cut off for depression of 12, and good sensitivity (78.6%) but lower specificity (64.4%), and an overall accuracy of 68.9% when compared to diagnosis by trained clinicians (28). The Cronbach's alpha values were 0.891 in a previous study in Rwanda (24), and 0.894 for the current study.

The Maternity Social Support Scale (MSSS), a modified version of the original scale by Webster and colleagues (29), was used to measure maternal social support. The original MSSS is a 6-item Likert scale that assesses support from spouses, friends, and family. This scale was expanded from 6 to 12 items to account for the cultural context of social support in Rwanda, including support from in-laws, community members, and neighbors. The MSSS has been previously used in research in Rwanda to measure social support in the perinatal period (14, 28). In the current study, a factor analysis identified two subscales: partner support (including three items such as my husband helps me a lot, there is conflict with my husband, I feel loved by my husband); and friend support (including items related to support from friends, neighbours, and the community), which were utilized to measure the level of maternal social support [detailed results reported in (30)]. The Cronbach's Alpha values were 0.775 for the partner support subscale, and 0.870 for the friend support subscale. The study collected socio-demographic characteristics such as age, marital status, education, occupation, and socioeconomic class. In Rwanda, socioeconomic class known as “ubudehe,” is a social stratification program that determines access to social welfare programs and is based on household income (31). The ubudehe system is divided into four groups, but none of the participants in this study fell into the highest category. Therefore, only the lower three categories are mentioned Obstetrics characteristics were also collected, including information on gestational age at birth (preterm or at term); pregnancy intention (whether the pregnancy was wanted), mode of previous deliveries (normal vaginal, vaginal with complications, cesarean section), and parity. Participants were asked to rate their general health on a 5-point scale ranging from very bad to excellent. These health scores were then recoded into a three-item scale indicating poor, neither poor nor good, and good.

Additionally, participants were asked to report any past exposure to negative life events, such asviolence or highly stressful experiences very (e.g., gender-based violence, childhood abuse, stranger violence, loss of any family member, etc.). This variable was recoded as a binary variable, distinguishing between participants who had never encountered any of these events and those who had experienced at least one stressful life event.

### Data collection process

The lead investigator provided training research assistants with backgrounds in midwifery and mental health nursing for data collection. These trained research assistants were introduced to midwives/nurses in charge of ANC at research sites. Through community health workers, researchers contacted potential participants and established a data collection schedule. Written informed consent was obtained from all participants, and for participants below 18 years of age, consent was also obtained from their legal guardians or parents (32).

### Data analysis

We used SPSS version 25 to analyse the data. The normality of continuous variables was visually assessed. Postnatal depressive symptoms (EPDS ≥12) were the binary primary outcome of interest. Univariate logistic regression analysis was conducted to explore potential correlations with all predictors, obtaining univariate odds ratios (OR) with 95% confidence intervals (CI) and *p*-values.

Variables with a bivariate relationship significance of *p* ≤ 0.30 were included in the multivariate logistic regression models using the Enter command. A relatively high significance threshold was set to account for the possibility that weakly correlated variables might have predictive power when analyzed together (33). The final multivariate model (adjusted model) was created using backward-stepwise regression, with a significance level of *p* < 0.05. The results are presented as adjusted odds ratios (AORs) with 95% CIs.

## Results

### Sample characteristics

A total of 396 individuals participated in the study, with 311 (78.54%) completing the follow-up until delivery, while 85 participants (21.46%) could not be reached via phone or through community health workers. The average age of the participants was 30.21 ± 6.61, with nearly half of them (47.90%; *n* = 149) aged between 24 and 34. The majority of participants (57.90%; *n* = 180) were legally married. Three-quarters of the participants (75.2%; *n* = 234) had only a primary education level or lower. Regarding occupation, most participants (80.1%; *n* = 249) reported engaging in farming or crop cultivation, and 53.1% (*n* = 164) belonged to the first and second (lowest) socioeconomic classes.

In terms of obstetric characteristics, the majority of participants (93.50%; *n* = 288) had term births, with 88.1% (*n* = 274) having given birth to one or three live neonates. Additionally, 75.60% (*n* = 235) reported that their pregnancy was wanted, while 71.40% (*n* = 222) had a normal delivery. Participants self-rated their general health, with 45% (*n* = 140) perceiving it as favorable. Moreover, 69.80% (*n* = 217) of the participants reported no extreme stressful or negative life events. Regarding maternal social support, 90.90% (*n* = 239) received good support from their partners, while 69.80% (*n* = 217) received good support from friends.

EPDS scores ranged from 0 to 30, with a mean of 7.75 ± 6.222. Participants with EPDS scores of 12 or higher were categorized as having postnatal depressive symptoms. For the overall sample, 20.9% (*n* = 65) of participants were found to have postnatal depressive symptoms.

### Bivariate relationships with postnatal depressive symptoms

In bivariate analyses, socioeconomic class was the only demographic variable significantly associated with postnatal depressive symptoms (OR: 0.36, CI: 0.16, 0.84, *p* = 002. In contrast to previous studies, age, marital status, occupation and education were unrelated to PND symptoms (see Table 1). Surprisingly, having no partner was not related to elevated PND symptoms.

**Table 1:** Social demographic distribution of participants by depressive symptoms.

	Depressive Symptoms (EPDS ≥12)			Univariate	
	Yes, _DP_ *N* (%) 65 (20.9%)	No _DP_ *N* (%) 246 (79.1%)	Total *N* (%) 311 (%)	Crude Odds Ratio (OR) (95% CI)	*p*-value
Age in Years (Mean: 30.21; SD: 6.61)					
24 or less	14 (21.50)	57 (23.20)	71 (22.80)	1	
25–34	30 (46.20)	119 (48.40)	149 (47.90)	1.03[0.51; 2.09]	0.94
35 or more	21 (32.30)	70 (28.50)	91 (29.30)	1.22[0.57; 2.62]	0.61
Marital status					
Single / Separated/ Divorced	13 (20.00)	35 (14.20)	48 (15.40)	1	
Married	36 (55.40)	144 (58.50)	180 (57.90)	0.67[0.32; 1.40]	0.29
Living together as spouses	16 (24.60)	67 (27.20)	83 (26.70)	0.64[0.27; 1.48]	0.30
Level of education					
No formal education	4 (6.20)	10 (4.10)	14 (4.5)	1	
Primary not completed	33 (50.80)	84 (34.10)	117 (37.60)	4.00[0.38; 42.37]	0.25
Primary completed	14 (21.50)	89 (36.20)	103 (33.10)	3.91[0.48; 31.91]	0.20
High School not completed	9 (13.80)	30 (12.20)	39 (12.50)	1.57[0.19; 13.26]	0.69
High school completed	3 (4.60)	20 (8.10)	23 (7.40)	3.00[0.31; 26.71]	0.33
Vocational education	1 (1.50)	3 (1.20)	4 (1.30)	1.50[0.14; 16.31]	0.74
Tertiary	1 (1.50)	10 (4.10)	11 (3.50)	3.33[0.16; 70.91]	0.44
Woman's occupation					
Farming/Cultural crops	54 (83.10)	195 (79.30)	249 (80.1)	1	
Employed	5 (7.70)	25 (10.20)	30 (9.60)	0.72[0.26; 1.97]	0.52
Not employed	6 (9.20)	26 (10.60)	32 (10.30)	0.83[0.31; 2.13]	0.70
Socio-economic class (Ubudehe) * 2 missing					
First category	12 (18.50)	21 (8.60)	33 (10.70)	1	
Second category	28 (43.10)	103 (42.20)	131 (42.40)	0.47[0.21; 1.08]	0.07
Third category	25 (38.50)	120 (49.20)	145 (46.90)	0.36[0.16; 0.84]	0.02

*p < 0.05, **p < 0.01 two tailed significance.

In terms of obstetrics, maternal and health variables, postnatal depressive symptoms were linked to gestational age at birth (preterm vs. term delivery) (OR = 0.35; CI = 0.14; 0.88, *p *= 0.03) and wanted pregnancy (OR = 2.39, CI = 1.33, 4.29, *p *= 0.004) but not parity or mode of delivery (see Table 2). Postnatal depressive symptoms were lower in participants with neither poor nor good (OR = 0.28, CI = 0.14; 0.57, *p *= 0.001) or good (OR = 0.90; CI = 0.40; 0.20, *p *= 0.001) health status, and higher in those who experienced stressful events (OR = 4.38; CI = 2.47, 7.77; *p *= 0.001), had poor partner support (OR = 7.41, CI = 3.06; 17.87, *p *= 0.001), or poor friend support (see Table 2).

**Table 2:** Association of maternal social support, obstetric characteristics, self-rated health status, and depressive symptoms.

	Depressive symptoms			Univariate	
	Yes, N (%) 65 (20.9%)	No, N (%) 246 (79.1%)	Total N (%) 311 (%)	Crude Odds Ratio (OR) (95% CI)	*p*-value
Age at birth *3 missing					
Preterm birth	8 (12.90)	12 (4.90)	20 (6.50)	1	
Term birth	54 (87.10)	234 (95.10)	288 (93.50)	0.35[0.14; 0.88]	0.03
Parity					
One to three	57 (87.7)	217 (88.2)	274 (88.1)	1	
Four and above	8 (12.3)	29 (11.8)	37 (11.9)	1.05[0.46; 2.42]	0.91
Pregnancy intention					
Wanted	40 (61.50)	195 (79.30)	235 (75.60)	1	
Unwanted	25 (38.50)	51 (20.70)	76 (24.40)	2.39[1.33; 4.29]	0.004
Mode of delivery					
Normal vaginal delivery	42 (64.60)	180 (73.20)	222 (71.40)	1	
Vaginal delivery with complications	6[9.20]	9 (3.70)	15 (4.80)	0.78[0.41; 1.48]	0.45
Caesarean section	17[26.20]	57 (23.20)	74 (23.80)	2.23[0.69; 7.17]	0.17
Perceived health status					
Poor	25 (38.50)	24 (9.80)	49 (15.80)	1	
Neither poor nor good	28 (43.10)	94 (38.20)	122 (39.20)	0.28[0.14; 0.57]	0.001**
Good	12 (18.50)	128 (52.00)	140 (45.00)	0.90[0.40; 0.20]	0.001**
Violence/negative life event (checked off at least 1)					
Yes	37 (56.90)	57 (23.20)	94 (30.20)	1	
No	28 (43.10)	189 (76.80)	217 (69.80)	4.38[2.47; 7.77]	0.001**
Maternal social support					
1. Partner support 48*Missing					
Good support	38 (73.10)	201 (95.30)	239 (90.90)	1	
Poor support	14 (26.90)	10 (4.70%)	24 (9.10)	7.41[3.06; 17.87]	0.001**
2. Friend support					
Good support	33 (50.80)	184 (74.08)	217 (69.80)	1	
Poor support	32 (49.20)	62 (25.20)	94(30.20)	2.88[1.64; 5.064]	0.001**

**p < 0.01 two-tailed significance.

### Multivariate prediction of postnatal depressive symptoms

Independent predictors of postnatal depressive symptoms that had a bivariate relationship significance of *p *≤ 0.30 were included in the multivariate logistic regression. Socioeconomic status, age, and pregnancy intention did not predict depressive symptoms and were not retained in the final model. The final model explained 0.35% of the variance. Postnatal depressive symptoms were significantly predicted by participants' perceived health status, social support, and prior exposure to negative life events (see Table 3). Specifically, participants who reported neither poor nor good health (AOR = 0.28, CI = 0.11–0.72, *p* = 0.007) or good health (AOR = 0.14, CI = 0.05–0.37, *p* = 0.001) were less likely to have scores in the depression range. Poor partner support (AOR = 4.22, CI = 1.44–12.34, *p* = 0.009) posed a high risk, while friend support (AOR = 0.47, CI = 0.23–0.98, *p* = 0.04) served as a significant protector. Participants who reported prior exposure to any violence or negative life events were two times more likely to develop postnatal depressive symptoms (AOR = 2.94, CI = 1.37–6.29, *p* = 0.005).

**Table 3:** Multivariate prediction of postnatal depressive symptoms among mothers in Rwanda.

	Full model			Reduced model		
	Odds Ratio (OR)	[95% CI]	*p*-value	OR	[95% CI]	*p*-value
Social economic classes						
First category	1					
Second category	1.86	[0.57; 6.04]	0.30	–	–	–
Third category	1.14	[0.51; 2.56]	0.75	–	–	–
Age at birth						
Term	1					
Preterm	3.37	[0.91; 12.53]	0.07			
Pregnancy intention						
Unwanted	1					
Wanted	0.47	[0.20; 1.11]	0.08			
Perceived health status						
Poor	1			1		
Neither poor nor good	0.29	[0.12; 0.73]	0.009**	0.28	[0.11; 0.72]	0.007**
Good	0.14	[0.05; 0.38]	0.001**	0.14	[0.05; 0.37]	0.001**
Violence/ negative life event						
No	1			1		
Yes	2.91	[1.35; 6.25]	0.006**	2.94	[1.37; 6.29]	0.005**
Maternal social support						
1. Partner support						
Good support	1			1		
Poor support	4.08	[1.38; 11.96]	0.01**	4.22	[1.44; 12.34]	0.009**
2. Friend Support						
Poor support	1			1		
Good support	0.47	[0.22; 0.90]	0.05*	0.47	[0.23; 0.98]	0.04*

*p < 0.05, **p < 0.01 two tailed significance.

## Discussion

The goal of this study was to explore the impact of social support on postnatal depressive symptoms among women in Rwanda. The findings revealed that one in five postnatal women in this sample reported postnatal depressive symptoms (20.9%), which aligns with similar research in low and middle-income countries (15). This underscores the significant burden of postnatal depression in the region and highlights the importance of identifying factors that can alleviate this burden. Partner support emerged as one of the most influential predictors of postnatal depressive symptoms, consistent with studies conducted in Uganda, Malawi, and Ethiopia (3, 12, 13). The literature suggests that the lack of support from a partner increases stress when raising the child alone (34) but it is not merely partner absence that matters, since individuals without partners in this sample did not report significantly higher risks of postnatal depressive symptoms in the bivariate analysis. It seems that a poor relationship with a partner plays a significant role on the mental wellbeing during this time (18). This implies that interventions focusing on strengthening and supporting partner relationships may be crucial in improving maternal mental health.

In addition to partner support, good friend support was associated with lower postnatal depressive symptoms, consistent with previous research in this area (2). The contribution of support from friends highlights the value of interventions utilizing peer support as a sustainable strategy for promoting maternal mental health in resource-limited settings where professional interventions may be less accessible. Similarly, although participants without partners did not emerge as being at particularly high risk in this sample, they have been found to have very elevated depressive scores in other studies in the Rwandan context (14). It should be noted that participants without partners were excluded from the multivariate analyses because they did not have scores on the partner support measure. Nonetheless, peer support may be a strategy to provide needed emotional, material and informational resources for those who lack a partner as well as for those whose partner relationships are poor.

It was perhaps not surprising to find that stressful life events were also one of the strongest predictors of elevated symptoms of postnatal depression. Intimate partner violence and other forms of abuse have consistently been reported as risk factors for both antenatal and postnatal depressive symptoms (15, 30). The impact of intimate partner violence aligns with the importance of good partner support. Additionally, previous mental health issues, which may be triggered or exacerbated by extreme negative events, are known to be associated with PND, emphasizing the significance of individual's personal history in assessing the risk of PND (18, 35). It is noteworthy that many of the economic and social factors typically associated with PND did not emerge as significant predictors in the multivariate or bivariate analyses. This could be attributed to the relatively low socioeconomic status of the sample, limiting the variance in this variable and reducing its predictive power in the study. It is also possible that participants facing the most challenging socioeconomic circumstances were less likely to participate likely to be traceable for the follow up survey to assess their postnatal scores. However, in the Rwandan context, perinatal women in the lowest category of socioeconomic status have access to additional resources, including health insurance and some social welfare support, which may have reduced the differences between them and those in the in higher categories.

Other common risk factors occurred with relatively low frequency, which could explain why they did not emerge as significant predictors. For example, there were few extremely young or old participants, few pre-term births, and relatively few with large families, limiting the possibility of finding significant relationships. Nevertheless, the presence of these risk factors as predictors in previous research conducted in Rwanda suggests the need for further exploration [e.g., (24)].

### Limitations

The study has certain limitations. The attenuated variance in some key predictors of PND within the sample may have limited the ability to identify relationships between risk and protective factors and elevated EPDS scores. Self-selection and selective attrition could have resulted in the exclusion of vulnerable or at-risk individuals from the study or the postnatal assessment. Moreover, women whith elevated EPDS scores were referred for mental health services but were still retained in the study. Although only a small number of women accepted treatment, this may have influenced the relationships between risk factors and EPDS scores.

## Conclusion

The high prevalence of postnatal depressive symptoms among postnatal Rwandan women stresses the urgent need for the integration of maternal mental health services into maternal and child health care across all levels of healthcare systems in Rwanda, and for midwives and nurses to provide a comprehensive assessment and early detection of depressive symptoms among women attending postnatal care or immunization programmes. The findings underscore the importance of assessing factors beyond obstetric and health-related aspects, including individuals' personal histories and current relationships, which may require additional skills and assessment strategies. The results also highlight the value of interventions aimed at strengthening partner and peer support, which can serve as low-cost and sustainable approaches in resource-limited settings (32, 36).

## Data Availability

The raw data supporting the conclusions of this article will be made available by the authors, without undue reservation.
